# Biomechanical Description of Zapateado Technique in Flamenco

**DOI:** 10.3390/ijerph18062905

**Published:** 2021-03-12

**Authors:** Wanda Forczek-Karkosz, Robert Michnik, Katarzyna Nowakowska-Lipiec, Alfonso Vargas-Macias, Irene Baena-Chicón, Sebastián Gómez-Lozano, Joanna Gorwa

**Affiliations:** 1 Department of Biomechanics, Faculty of Physical Education and Sport, University of Physical Education in Krakow, 31-636 Kraków, Poland; wanda.forczek@awf.krakow.pl; 2 Department of Biomechatronics, Faculty of Biomedical Engineering, Silesian University of Technology, 44-100 Zabrze, Poland; Robert.Michnik@polsl.pl (R.M.); Katarzyna.Nowakowska-Lipiec@polsl.pl (K.N.-L.); 3 Telethusa Centre for Flamenco Research, E-11004 Cádiz, Spain; vargas@flamencoinvestigacion.es; 4 Department of Flamenco Dance, Pepa Flores Professional Dance Conservatory, E-29007 Málaga, Spain; irenecpd@gmail.com; 5 Performing Arts Research Group, San Antonio Catholic University of Murcia, E-30107 Murcia, Spain; sglozano@pdi.ucam.edu; 6 Department of Biomechanics, Chair of Theory and Methodology of Sport, Faculty of Sport Sciences, Poznan University of Physical Education, 61-871 Poznań, Poland

**Keywords:** technique, footwork, intangible cultural heritage, flamenco, zapateado

## Abstract

The main purpose of this study was to identify a dancer’s body alignment while performing flamenco footwork to provide a detailed description that could be used by flamenco practitioners: teachers, instructors and students of different levels of advancement. The zapateado technique performed by a professional flamenco dancer was analyzed. The biomechanical analysis was based on 30 cycles composed of six repeating sequences of strikes. Kinematic recordings were performed using a Vicon system, while the measurement of the ground reaction forces (GRF) was accomplished with a Kistler force plate. The following parameters were analyzed: the time of each foot strike, the maximal value of the vertical component of GRF normalized to body weight (BW) for subsequent footwork steps, the impulse of the GRF and the kinematics of pelvis and lower limb joints, and an exemplary waveform view of the sound of footwork strikes was shown. The average values of the vertical component of GRF ranged between 0.6 and 2.7 BW. The maximal anterior pelvic tilt was 29°, with a 6° range of motion (RoM). This mobility was accompanied by 20° hip RoM and by ~40° knee RoM throughout flexion. The conclusions provide practical information that a teacher and flamenco student should receive.

## 1. Introduction

Intangible cultural heritage (ICH) refers to oral traditions, performing arts, rituals, knowledge, traditional crafts and festive events. This kind of heritage is transmitted through the knowledge and skills from one generation to the next and is an important factor in maintaining cultural diversity [1].

Flamenco is among the most characteristic elements of Spanish culture which belongs to the ICH. Considering its popularity, in Spain alone, there are about 300 academies, conservatories or centers for teaching flamenco dance. The highest density of practitioners is observed in four regions out of 17. These are the following: Andalusia, Murcia, Madrid and Catalonia. Flamenco is also very popular around the world [2,3], and it can be danced by itself, or some of its elements (e.g., different footwork) can be applied to other dances, e.g., Paso doble, which belongs to the ballroom dancing, Latino American style.

There are many different styles (palos) within flamenco music, each with its own characteristic compás. This is the rhythmic pattern which the dancer must follow while striking the ground. As for the dance, the compás is as important as the aesthetics of movements in flamenco. Therefore, the teachers, especially in Spain, insist on the importance of listening to the rhythm. Another aspect that also concerns the musicality is the soniquete and the subtlety of the sounds (musicality) in performing the footwork [2]. The sounds that the dancer makes also depend on the intensity with which she or he hits the ground, which is related to the ways of beating the ground, e.g., with the planta (the forefoot), the golpe (the whole foot) or the tacón (the heel). In general, learning the complex skills involved in dance presents many challenges. It is a continuous trial-and-error process, which is fraught with difficulties [4]. So, we must ask the following question: what really matters during the mastery of flamenco dance?

Flamenco, as one of the most recognizable elements of Spanish culture, has become a worldwide phenomenon, and was placed on the ICH list in 2010. Flamenco art was formed by the cultural interaction between several nations settling in the south of Spain and it combines three genres: traditional singing, guitar playing and dancing. The phenomenon of flamenco is based on the personal contact of a teacher (master) and student. The spirit and philosophy of flamenco must be passed on to the next generation. However, dances can also be taught in various ways, such as textual, video and graphic notation [5].

Flamenco dance is not only spiritual and very emotional, but it is recognized as a very demanding dance technique where the rhythm plays an essential role. Even apparently simple technical elements are the result of the dynamic interactions between different segments of the body. Among the most fundamental movements in flamenco is footwork (zapateado) performed in traditional high-heeled shoes. Zapateado is a repetitive percussive footwork with metatarsus, flat foot and heel [6]. Therefore, one can say that flamenco dancers act on a floor like a drummer [7], and flamenco itself is called a percussive dance. The percussive footwork during dancing generates a series of shock waves, which impose unusual demands on the musculoskeletal system [8]. The foot is the first organ which has to deal with the heel and/or metatarsal impact-generated shock waves. The external environment of the foot, e.g., the shoes and the floor, has a great importance to attenuate shock waves to some extent [8]. However, the natural mechanisms of shock cushion are provided by the proper alignment of the body. Attenuation of the shock waves takes place mainly in the joints, and increased muscular activity is needed to stabilize them [9]. Participants showed a higher incidence of functional disorders and injuries in the knees, lumbar and cervix, and perhaps this difference is because the flamenco footwork technique requires a semi-flexion of knees, which does not integrate vertical jumps or tilts; these would enable the vibrations causing an impact on the spine to be dissipated [10].

Due to technological advancement in recent decades, we have gained insight into body movements during dancing. Biomechanics is a tool which provides information on human movement, but it also helps to understand forces acting on the body and interactions that take place within the body segments [11]. However, bearing in mind the results of Forczek et al. [12], the instrumentation involved in biomechanical investigations of flamenco is very simple; thus, some technical issues still need to be solved.

Considering comprehensive scientific analyses of the technical performance of various dance styles, such as the classics [13,14,15], modern dance [16,17,18], Irish dance [19] and a whole range of folk dances from various regions of the world [20], there is a lack of a detailed technical analysis of flamenco dance in the scientific literature [12].

The main purpose of this study was to identify the dancer’s body alignment while performing flamenco footwork, to provide a detailed description that could be used by flamenco practitioners: teachers, instructors and students of different levels of advancement. This is the first study to provide a comprehensive approach to simultaneously present the results of kinematics, dynamics and audio signals generated during zapateado, one of the most typical sequences of footwork in flamenco choreography.

## 2. Materials and Methods

Our research comprised a synchronized registration of kinematic and dynamic variables together with audible signals generated by feet during zapateado.

### 2.1. Ethics Statement

The study received the approval from the Ethics Committee of the San Antonio Catholic University of Murcia, Spain (number: 6777.2017), and the research was conducted according to the ethics principles stated in the Helsinki Declaration. The flamenco dancer gave informed consent to participate in the research.

### 2.2. Materials

Biomechanical and acoustic analyses were based on 30 cycles composed of 6 repeating sequences of strikes executed with the right and left leg (as a whole, 180 elements) performed by a professional flamenco dancer (34 years; height 1.65 m; mass 58 kg; BMI 21.30 kg·m^−2^). She had been practicing flamenco for 31 years (16 years as a professional).

### 2.3. Biomechanical Registration

The task of the subject was to perform ZAP 3 [21]: a sequence of 6 footwork steps with the right and the left foot (see the detailed description of ZAP 3 test in the Results section). The duration of the test was 15 s. Every footwork step is made in order to provide the greatest possible sound, performed as quickly as possible and with a melodic rhythmic sound. The test was performed using the specific high-heeled shoes (6 cm tall) twice: 1. when the right foot (the dominant one) was on a force plate; 2. when the left foot (nondominant) was placed on a force plate.

### 2.4. Data Recording and Analysis

Kinematic recordings were performed using a 5-camera video-based (120 Hz sampling rate) motion capture system (Vicon 250; Oxford Metrics Ltd.; Oxford, UK), while measurement of the ground reaction forces (GRF) was accomplished with a Kistler force plate (1000 Hz sampling rate). Thirty-five infrared reflective markers were attached to the subject’s body according to the Golem set-up: 4 were placed on the head, 4 on the trunk, 3 on the pelvis, 7 on each of the upper and 5 on each of the lower limbs.

Audible signals generated by the feet during zapateado were recorded with the Casio EXILIM High Speed EX-ZR1000 camera, 240 fps.

The subject was asked to perform test ZAP-3 twice, in order to place both the right and left feet on the force platform. Prior to the test, we identified the right leg as the dominant for the dancer [22].

### 2.5. Analyzed Variables

**Figure 1:**
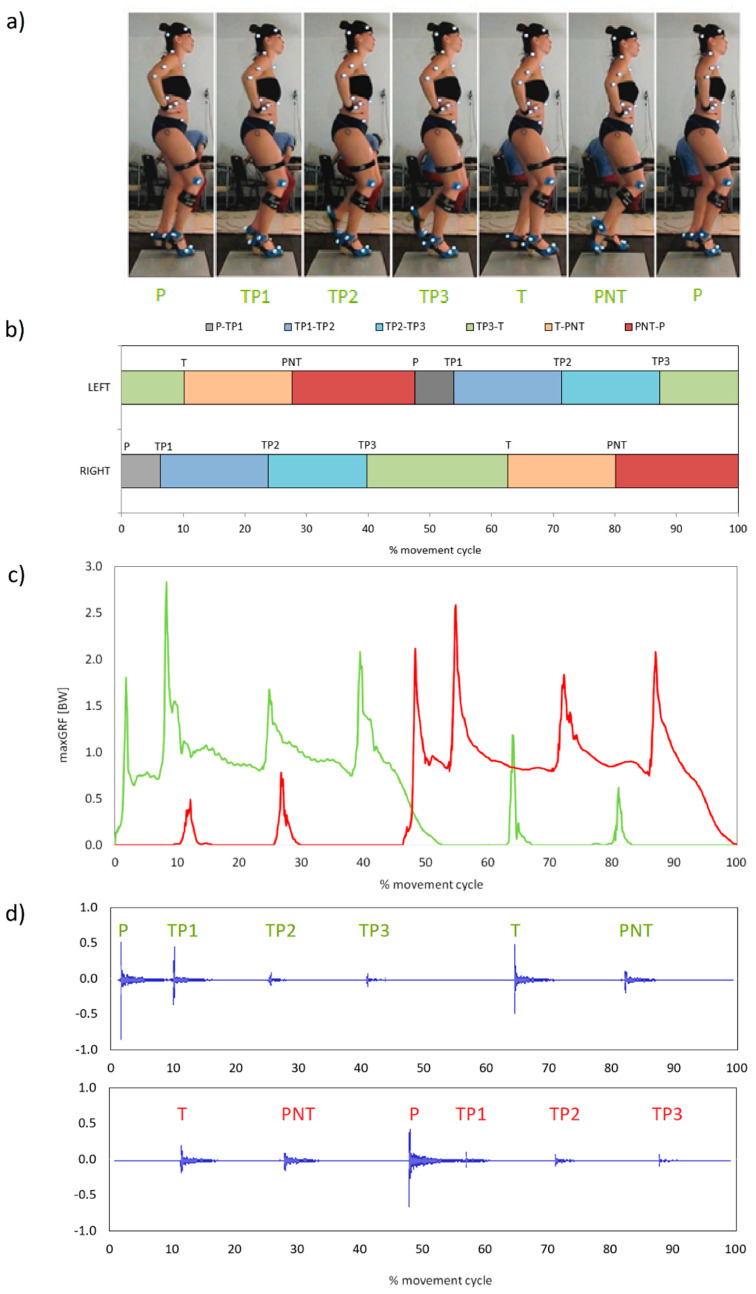
ZAP-3 cycle in flamenco: (**a**) body position; (**b**) averaged duration between successive strikes; (**c**) exampled course of the vertical component of ground reaction force (GRF); (**d**) example waveform view of the sound of footwork strikes.

All kinematic, dynamic and acoustic quantities are presented in successive cycles defined as the time between two consecutive Planta strikes (P) performed with the same limb (Figure 1a) during the ZAP-3 test. A single cycle consisted of 6 consecutive foot strikes. 

The following parameters were analyzed:

- Time of each foot strike during ZAP-3 test—*t_GRF_* [s];
- Maximal value of the vertical component of ground reaction force (*GRF*) normalized to body weight (BW) for subsequent footwork steps—maxGRF [BW];
- Loading rate of the ground reaction force for subsequent footwork steps—*LR_GRF_* [BW]:
  (1) $$LR_{GRF}=\frac{maxGRF}{t_{GRF}}$$
- Kinematics of pelvis and lower limb joints during ZAP-3: pelvic obliquity, pelvic tilt, pelvic rotation, hip abduction–adduction, hip flexion–extension, hip rotation, knee flexion–extension, dorsi–plantarflexion and inversion–eversion in ankle joint.

In addition, it was decided to conduct a qualitative analysis of an exemplary waveform view of the sound of footwork strikes during ZAP-3.

### 2.6. Statistical Analysis

For the analysis of the ground reaction force, the following descriptive statistics were used: mean, standard deviation, maximal and minimal values. The normality of distribution for all variables was checked with the Shapiro–Wilk test. The differences between the values of the analyzed parameters for 6 different foot strikes during the ZAP3 test (P, TP1, TP2, TP3, T, PNT) was assessed by ANOVA. When significant differences were detected, post-hoc tests were performed by means of the Conover–Iman test. Statistical analyses were performed using the statistical package Statistica 13.1 (StatSoft). Reported results are considered significant for *p* ≤ 0.05.

## 3. Results

### ZAP-3 Test Description

The first footwork step is called **Planta (P)** in flamenco jargon. It is made by the supporting leg. However, the supporting role changes from one limb to another. This transfer of the body weight to the second limb follows rapidly and is initiated before P occurs and terminated when it is completed. At the instant of the foot strike, the knee is flexed and extended, and the impact of the floor contact is generated by the forefoot, provided by all the metatarsal heads. When the limb goes to the posterior plane, the ankle is relaxed but with the foot in a slight plantar flexion. This leg takes the responsibility of support while preparing to perform the second footwork step that will be produced by dropping the heel on the floor. This is called **Tacón de Planta (TP1)**. The strike is made with the forefoot on the ground. The hindfoot is raised proximally to gain momentum and to hit the ground making an audible sound. Just after the other leg performs its footwork, called Tacón (T), the supporting leg repeats a **Tacón de Planta (TP2)** when the forefoot is planted. The last step provided in this position by the supporting leg, whilst the forefoot is planted is **Tacón de Planta (TP3)**, when the heel drops to the floor following the second leg strike Punta (PNT).

The fifth footwork of the right limb (the nonsupporting one) is provided by the heel impact, the so called **Tacón (T)**, on the floor to make the sound in front of the subject. This happens with flexing and extending the knee joint and foot in dorsiflexion.

**Figure 2:**
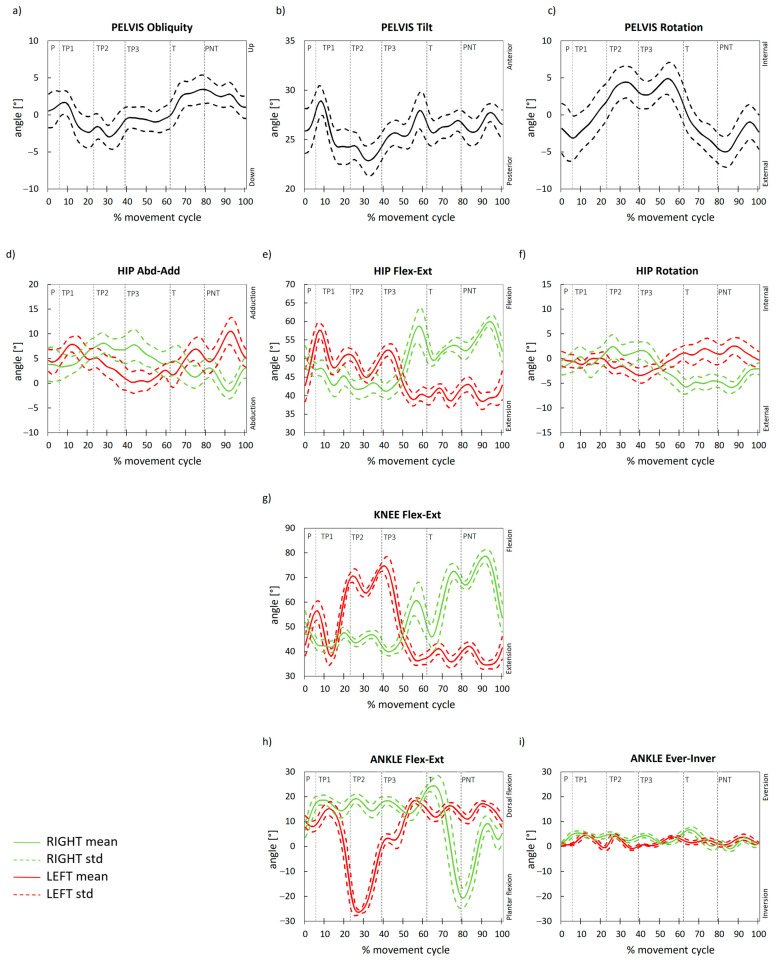
Kinematics of body movement in Zap-3 test: (**a**) pelvis obliquity, (**b**) pelvis tilt, (**c**) pelvis rotation, (**d**) hip abduction–adduction, (**e**) hip flexion–extension, (**f**) hip rotation, (**g**) knee flexion–extension, (**h**) ankle flexion–extension, (**i**) ankle eversion–inversion.

The last one is also performed by flexing and extending the knee, which is hardly lifted from the ground towards the posterior plane because the impulse to hit the ground backwards is enough. The foot is in plantarflexion, and the strike is provided by tapping the tip of the toe on the floor behind the base of support. This is called **Punta (PNT)** (Figure 1 and Figure 2, Table 1).

Friedman’s ANOVA test revealed statistically significant differences in the vertical maxGRF and impulse of force LR_GRF_ between the successive foot strikes (P, TP1, TP2, TP3, T, PNT) during ZAP3 (*p* ≤ 0.05). Post-hoc tests showed that maxGRF values are significantly different for almost every pair of strikes. There were no differences between the maxGRF obtained for P and TP3, and between T and PNT for the left lower limb. Statistically significant differences were also noted for the values of the loading rate of the ground reaction force between the majority of the analyzed pairs of strikes (Table 2).

## 4. Discussion

The main purpose of our study was to document the technique of footwork (zapateado). We provided a detailed description of the most common set of steps used in choreography. Due to the repetitive stamping of the feet in high-heeled shoes against the floor, flamenco is called a percussive dance where the dancer is a musician—a percussionist, who uses his or her body as an instrument to make different sounds and rhythms by means of the feet beating the ground, hands clapping together or on the body [2].

### 4.1. Kinematics

#### 4.1.1. Initial Body Position—How to Develop Flamenco Dance Posture

The impressive attitude and the true spirit of flamenco are reflected in the good posture of the dancer. This will allow greater freedom of movement for the arms, head, trunk and feet for footwork. It will also improve his or her confidence. Therefore, while standing, there is a need to separate the upper body from the lower body at the hips. To hold the chest proudly, it should be actively lifted away from the hips; relaxed shoulders will allow the shoulder blades to rest lightly on the rib cage. The head is positioned on an extended neck looking straight forward, not down at the floor or at the feet (Figure 1a).

#### 4.1.2. Body Alignment during Footwork

The percussive stamping of the feet produces whole-body vibrations, which must be attenuated by the joints and muscles of the legs to a frequency that will not cause bodily harm [8]. The full intensity of the floor impact is reduced by shock-absorbing reactions at the ankle, knee and hip. As our registrations revealed, the average values of the vertical component of GRF ranged between 0.6 and 2.7 BW executed with appropriate loudness (Figure 1a,c). Thus, among the key elements while performing zapateado is a proper configuration of the lower limb joints and relevant activity of the muscles stabilizing them [9]. “The lower limbs, their muscles and the abdominal part of the body are mostly responsible for a successful implementation of the tasked dance technique. The ability to contract them swiftly and to relax them determines the necessary dynamics of the dance performance, the ability to react timely with specific muscle groups influences rational utilization of the dancer’s energy, as well as the very appearance of muscles largely contribute to the esthetic expression of the move performance.” [23].

Proper positioning with flexed hips and knees, a straight back and feet in line with the hips (Figure 1a) may provide effective mechanisms of shock cushioning. In between there is the pelvis with a dual task: first, it is a link between the lower limbs and, second, it serves as the bottom of the upper body that rides on the hip joints [24]. In our footwork test performed in high-heeled shoes, the maximal anterior pelvic tilt is 29°, with a 6° range of motion (RoM). This mobility is accompanied by 20° hip RoM throughout flexion. The knee stays at the flexion RoM (~40°). 

Considering an ergonomic adaptation following fast footwork, it is mostly the role of the knees that may dampen the impact of the feet–ground contact up to five times [25]. The knee joint, due to the spatial segment orientation of the femur and tibia, is in the best position of all the lower limb joints to help attenuate the vertical GRF [26]. Therefore, Echegoyen et al. [6] emphasized the problem in Spain where teachers instruct the students to perform zapateado without knee flexion or with minimum flexion of the knee. When the knee is extended, it produces less absorption through its musculotendinous system, increasing the force transmitted to the passive structures of the knee and joints above positioned [6,27]. Additionally, the dynamic loading in flamenco footwork has an unusual pattern due to the traditional high-heeled shoes worn by the dancer [8].

The control of the pelvis position during zapateado requires isometric activity of the surrounding muscles, which results from the necessity of stabilizing the trunk and pelvis. In general, we observed limited spatial oscillations of the pelvis in our subject (Figure 2b). According to Haight [28], the greatest “back-up” for the dance includes strong lower limbs and strong muscles of the trunk. Movements of the pelvis are restrained by the hip muscles, while the back and abdominal musculature control the alignment of the trunk over the pelvis. Positions and movements involving end-range extension are an integral part of the dance aesthetic. The biomechanical analysis of the human locomotor system revealed that unfavorable situations are those when muscles are subjected to long isometric tension and when muscles stabilize the multisegment biomechanism bases [14]. It seems that the flamenco falls well within the scheme of the aforementioned static loads. Flamenco aesthetics require the control and immobility of the trunk and neck during the zapateado and a sustained spinal position close to the functional limit [7,29]. This causes a stiffness that could cause overloads in the back muscles. The use of the abdominal muscles and hip extensors (buttocks) is of great importance in the correct pelvis stabilizing process [14]. 

Abdominal muscles stabilize the trunk and limit the anterior pelvic tilt which, if exaggerated, is adverse. The results of the Gorwa et al. (2020) study indicate that at least the effectiveness of exercises aimed at the engagement of the abdominal muscles in keeping the pelvis stable in the sagittal plane should be evaluated and monitored in young dancers [14]. These muscles will have the same importance in flamenco, where the main movement task is played by the lower limbs with a stabilized pelvis. Wilmerding et al. [29], whose study was to determine the degree and magnitude of changes of angulation in pelvic tilt in young dancers, showed that 4 of the 16 subjects practicing flamenco showed increased anterior pelvic tilt, which correlates with lumbar lordosis. An increase in lumbar lordosis would generally be considered the biomechanically preferable accommodation of the spine to assist in absorbing the greater vertical shock loading [30]. Deepened lumbar lordosis causes high shear forces on the intervertebral discs and posterior parts of the vertebrae. Expert dancers differed from novice dancers in terms of postural pelvic control, suggesting that the control of the pelvis requires extended practice [31]. The examination of trunk coordination and postural control may be important in determining movement patterns associated with high levels of skill [32].

#### 4.1.3. Detailed Description of the Locomotor Activity during ZAP-3

During zapateado, many patterns of body part movements and muscle control are involved. At the moment the foot strikes the ground, the limb should be optimally positioned to absorb some of the shock of the floor contact while preserving postural stability and performing footwork with appropriate loudness and musicality. Thus, characteristic positions for each joint during the specific footwork strike are presented below. Since qualitative analysis revealed a huge similarity in the movement performances of the right and left lower limbs, our description is related to the right limb quantities.

During P footwork, the pelvis is anteriorly tilted (~26°), and the hip joint is flexed (~50°), accompanied by the knee flexion (~50°) and ankle plantar flexion (~7°). In TP1, the pelvic anterior tilt increases to reach 29°, and afterwards, it decreases in TP2 and TP3 when it oscillates between 23° and 25°. The hip slightly extends (till ~46°) and fluctuates within 46°–42° in TP2 and TP3 strikes. At the same time, the knee reduces its flexion to 43°, and throughout TP2 and TP3, when the heel rises, knee flexion oscillates between 40° and 49°. Plantar flexion is a part of the primitive flexor synergy (it accompanies hip and knee flexion). The increase in plantar flexion in TP1 to 19° is kept within small fluctuations throughout the next two strikes (TP2, TP3). T footwork is preceded by unloading weight from the limb, lifting the foot from the ground and moving the limb forward to strike the floor with the heel. It is accompanied by a sudden increase in anterior pelvic tilt (28°), which oscillates between 28 and 26° throughout PNT until the next P is initiated. Maximal pelvic anteversion before T is accompanied by larger hip flexion (~60°), which reduces its flexed posture after the impact (to ~51°) and remains stable in PNT. Then, it reaches its maximum (~62°), preparing the limb for another cycle of footwork steps. Similarly, just before the T strike, the knee joint flexion increases (to ~60°) to advance the limb and perform this strike in front of the body. While hitting the ground, the knee extends to ~46° and then again visibly flexes to strike the floor with the toes in PNT. Afterwards, knee flexion is diminished to prepare for another P footwork. The ankle joint continues to plantarflex, reaching its peak during T (~26°). Then, a decrease in plantar flexion occurs in order to reach maximal dorsiflexion in PNT (~20°). Afterwards, the foot is prepared for P footwork. 

To understand how much the flamenco dancer’s body is used during the footwork, we consider the locomotor function during walking, the human basic daily activity. The pelvis has an anatomical 10° anterior pelvic tilt. During typical stepping in walking, an additional 4° pelvic tilt occurs, and its entire range of movements is 4° [24]. While in our footwork test, the maximal anterior pelvic tilt is 29°, with a RoM of 6°, this mobility is accompanied by 20° of the hip RoM throughout flexion. Hip flexion allows the pelvis and trunk to remain erect. The normal range during free walking for the hip is 40°. The exchange of motion from flexion to extension is gradual. It ranges from 30 flexion to 10° extension. The knee also stays at the flexion range of motion (~40°). Partial knee flexion serves to compensate for the elevation of the heels [29], and it contributes to controlled shock absorption. Normal knee motion during walking represents greater and lesser degrees of flexion within the full range of 0° to 60°. Faster walking speed is associated with greater knee flexion (especially in loading response). The most mobile element of the locomotor unit is the ankle joint with a 42° entire range of motion during the footwork; however, in everyday activities, the RoM required in the sagittal plane is significantly reduced, with a maximum of 25° for walking.

Simultaneously, the coronal and transverse plane motions of the pelvis are followed by the hip joint. In general, the pelvis reveals small oscillations (~6°) in the frontal plane, while in walking, its range of motion is 8°. During weight acceptance in walking, the contralateral side of the pelvis drops on average by 4° in the coronal plane. In pre-swing, the ipsilateral pelvis drops by 4° as the contralateral limb abductors yield under the high demand of loading response. However, in a transverse plane, its RoM (~10°) is similar to gait oscillations (5° forward and 5° backward). Maximum forward rotation contributes to the step length of the lead limb. Maximum backward rotation contributes to a trailing limb posture.

For normal foot motion, sagittal and frontal planes of motion must be smooth to provide joint stability. The majority of ankle eversion/inversion occurs at the subtalar joint. While in walking, its range of motion in the coronal plane is 6.3° eversion and 8.3° inversion [33]; our subject revealed ~10° (8° eversion/2° inversion). During gait, the movement of the foot is synonymous with the movement of all the bones of the lower extremity. As an intricate mechanism that cushions the body and adapts to uneven surfaces, the foot provides traction for movement, awareness of joint and body position for balance and leverage for propulsion [34].

#### 4.1.4. Feet during Zapateado

Dancers are skilled athletes who perform within a unique set of movement constraints [21,35,36]. In flamenco, footwork needs to be performed very fast with high precision, stabilizing the trunk and facilitating arm movements at the same time. The dancer in our study achieved a mean frequency of 11.8 footwork steps/s. Movements of the feet are of great importance: any strike with any part of the foot is not an act of force [25]. It requires some know how with regard to how to strengthen the strike. The movement should be performed dynamically—the foot goes downward. Flamenco dancing is concentrated downward toward the ground, the most intense energy on the spot. The foot is the “root” between the body and the earth. Any contact with the ground performed with any part of the foot during zapateado should be short (see Table 1). Then, the high impulse of force ensures a dynamic and fast displacement of the foot—following the 2nd law of Newton. We achieved the highest values of the loading rate for the most dynamic vertical loads. Flamenco dancers wear high-heeled shoes and perform fast footwork involving concentric contractions of both the plantar flexors and dorsiflexors (in our case within 42° range of movement). The change of the ankle position may decrease lateral ankle stability; thus, the high frequency of footwork is due to a very limited displacement of the feet. If the foot performing any kind of strike goes too far, it will need to cover a longer distance. So, when the foot is far from the body line, although its mass is merely 1.4% BW [37], moment of inertia of the whole limb increases. The longer time required to displace the foot affects the quality and quantity of footwork frequency and its “soniquete” and may lead to premature fatigue. It may disturb the stability of the body and center of mass (COM) displacements. The proper position of the lumbar lordosis helps the pelvis stabilize and distribute the body weight over the lower limbs, as it assists in positioning the trunk’s center of mass above the hips [30]. Regarding the smooth oscillations of the pelvis segment in our study, we may assume that the COM position remains stable. Struggling with stability is very demanding in terms of muscular activity, which is why the movements performed are far from ergonomic. Additionally, any muscular imbalance may affect the body’s ability to dampen the vibrations. To prevent that, a supporting leg should be loaded, with the pelvis stabilized to control the upper part of the dancer’s body and reduce unnecessary movements.

### 4.2. Kinetics

The traditional high-heeled shoes used by flamenco dancers do not provide adequate shock absorption [8,38]. Thus, when striking the ground, dancers are subjected to serious loads on the structures of the supporting limb due to high peaks of force that are several times their body weight [6,19]. As our registrations revealed, the average values of the vertical component of the ground reaction force ranged between 0.6 and 2.7 BW. When the forefoot strikes the ground (P), it reaches double the body weight. The impact force initiates rapid heel drop (TP1), which generates a spike 2.7 times the BW. This is followed by TP2 and TP3 when the forefoot is placed on the ground and the heel strikes the floor. The highest impact in both footworks reaches twice the BW. Shortly after the rapid transfer of the body weight to the second limb, the heel strikes the floor (T) in front of the body and provides a spike of the vertical component of GRF equal to the BW. Finally, the lowest impact performed with the toes accompanies PNT (0.6 BW).

Additionally, during gait, the magnitude of the vertical component of GRF varies with the changing limb position. There are two peaks generated during the stance phase (~110 BW each) separated by the valley (~80% BW) [24]. Zapateado is performed to produce sound with appropriate loudness and musicality. This in turn is provided due to striking the ground with the given part of the foot while the body is kept in the right position. By qualitatively analyzing the sound wave recorded in a single ZAP-3 cycle (Figure 1d), it can be seen that the highest values of sound volume occurred for those foot impacts on the ground for which the vertical component of the ground response was the highest (P, TP1).

### 4.3. Direction for Future Research

Bearing in mind that biomechanical analysis is focused not only on the forces acting on the body, the kinematics of the movements, but also on muscle activity, our future study should also involve electromyography in flamenco investigation. This will help to understand how the nervous system plans and controls impact absorption, which is essential for designing a strategy to prepare the muscles to absorb an impact.

## 5. Conclusions

This is the first study which comprehensively assesses the body posture of a flamenco dancer while performing footwork. We provided an explanation from a biomechanical perspective of the meaning of coordinated work of the body segments aimed to generate rhythmic patterns of strikes. We hope our results can help flamenco practitioners verify their intuitive body arrangement while dancing.

### Take Home Message

Flamenco dance teaching requires fine tools for the training of the dance artist but at the same time should provide some precautions to reduce the risk of overloading and injuries. Dance educators and instructors can derive from this research a safer and more effective technique to impart to students.

1. Start with slow speed: slowly practice single elements before you join everything together to move your whole body.
2. When increasing speed, practice footwork only at the maximum speed at which you can still perform each foot strike accurately and with an audible sound.
3. Pay special attention to the alignment of your whole body. Due to the proper position of the body, the dynamic, smooth and rhythmic performance of footwork will be safe for the dancer.
4. Follow the compás as the most important quality.
5. Use your pelvic stabilizers (abdominal and hip muscles) consciously.

## Figures and Tables

**Table 1:** Maximal value of the vertical component of ground reaction force and the value of loading rate of ground reaction force (GRF) normalized to body weight for the ZAP-3 test.

		maxGRF [BW]			LR_GRF_ [BW/s]		
		Mean ± std	min–max	Shapiro–Wilk Test (p)	Mean ± std	min–max	Shapiro–Wilk Test (*p*)
**RIGHT**	**P**	2.22 ± 0.60	0.84–2.98	**0.48**	115.11 ± 52.37	33.74–241.98	**0.28**
	**TP1**	2.71 ± 0.23	2.32–3.11	**0.97**	260.94 ± 66.23	159.35–387.01	**0.94**
	**TP2**	1.91 ± 0.42	1.35–3.11	0.01	166.98 ± 102.70	36.21–319.03	**0.17**
	**TP3**	1.88 ± 0.47	0.91–2.30	0.02	152.39 ± 62.70	55.87–230.35	**0.12**
	**T**	1.06 ± 0.30	0.34–1.38	**0.09**	113.88 ± 69.61	24.33–308.22	0.04
	**PNT**	0.59 ± 0.23	0.19–0.94	**0.86**	78.69 ± 48.76	6.95–186.55	**0.82**
**LEFT**	**P**	2.35 ± 0.42	1.46–3.08	**0.82**	117.77 ± 34.11	69.53–205.48	**0.17**
	**TP1**	2.71 ± 0.35	2.19–3.60	**0.15**	243.47 ± 87.74	95.09–414.82	**0.53**
	**TP2**	1.99 ± 0.14	1.69–2.20	**0.95**	96.78 ± 34.96	60.17–164.72	0.02
	**TP3**	2.42 ± 0.31	1.93–2.93	**0.81**	185.45 ± 37.15	137.88–253.65	**0.25**
	**T**	0.62 ± 0.24	0.24–1.01	**0.68**	47.02 ± 22.15	22.3–87.60	0.04
	**PNT**	0.65 ± 0.19	0.26–0.94	**0.81**	61.67 ± 21.95	19.96–111.24	**0.87**

The *p*-value for normal distribution is in bold; maxGRF—maximal value of the vertical component of ground reaction force; LR_GR_—value of loading rate of GRF force; BW—body weight; std—standard deviation. Footwork steps during ZAP-3: P—Planta; TP1, TP2, TP3—Tacón de Planta; T—Tacón; PNT—Punta.

**Table 2:** Conover–Iman post-hoc test results for maxGRF and LR_GRF_ for subsequent foot strikes in the ZAP3 test.

**maxGRF**						
	**RIGHT**					
	**P**	**TP1**	**TP2**	**TP3**	**T**	**PNT**
**P**		<0.05 *	0.02 *	0.09	<0.05 *	<0.05 *
**TP1**	<0.05 *		<0.05 *	<0.05 *	<0.05 *	<0.05 *
**TP2**	0.02 *	<0.05 *		0.52	<0.05 *	<0.05 *
**TP3**	0.09	<0.05 *	0.52		<0.05 *	<0.05 *
**T**	<0.05 *	<0.05 *	<0.05 *	<0.05 *		0.01 *
**PNT**	<0.05 *	<0.05 *	<0.05 *	<0.05 *	0.01 *	
	**LEFT**					
	**P**	**TP1**	**TP2**	**TP3**	**T**	**PNT**
**P**		<0.05 *	<0.05 *	0.19	<0.05 *	<0.05 *
**TP1**	<0.05 *		<0.05 *	<0.05 *	<0.05 *	<0.05 *
**TP2**	<0.05 *	<0.05 *		<0.05 *	<0.05 *	<0.05 *
**TP3**	0.19	<0.05 *	<0.05 *		<0.05 *	<0.05 *
**T**	<0.05 *	<0.05 *	<0.05 *	<0.05*		0.6
**PNT**	<0.05 *	<0.05 *	<0.05 *	<0.05*	0.6	
**LR_GRF_**						
	**RIGHT**					
	**P**	**TP1**	**TP2**	**TP3**	**T**	**PNT**
**P**		<0.05 *	0.16	0.16	0.7	0.07
**TP1**	<0.05 *		<0.05 *	<0.05 *	<0.05 *	<0.05 *
**TP2**	0.16	<0.05 *		1	0.07	<0.05 *
**TP3**	0.16	<0.05 *	1		0.07	<0.05 *
**T**	0.7	<0.05 *	0.07	0.07		0.16
**PNT**	0.07	<0.05 *	<0.05 *	<0.05 *	0.16	
	**LEFT**					
	**P**	**TP1**	**TP2**	**TP3**	**T**	**PNT**
**P**		<0.05 *	<0.05 *	<0.05 *	<0.05 *	<0.05 *
**TP1**	<0.05 *		<0.05 *	0.31	<0.05 *	<0.05 *
**TP2**	<0.05 *	<0.05 *		<0.05 *	<0.05 *	<0.05 *
**TP3**	<0.05 *	0.31	<0.05 *		<0.05 *	<0.05 *
**T**	<0.05 *	<0.05 *	<0.05 *	<0.05 *		0.42
**PNT**	<0.05 *	<0.05 *	<0.05 *	<0.05 *	0.42	

* *p* ≤ 0.05; maxGRF—maximal value of the vertical component of ground reaction force; LR_GRF_—value of loading rate of GRF force; BW—body weight; std—standard deviation. Footwork steps during ZAP-3: P—Planta; TP1, TP2, TP3—Tacón de Planta; T—Tacón; PNT—Punta.

## Data Availability

The datasets used and/or analyzed during the current study are available from the corresponding author on reasonable request.
